# Correlation Between Coronary Artery Ectasia and Past Stressful Life Events and Psychological Hardiness: A Cross-Sectional Analysis

**DOI:** 10.3390/medicina62030507

**Published:** 2026-03-10

**Authors:** Hasan Korkmaz, Irfan Yaman, Guney Sarioglu, Muhammed Fatih Tabara, Esengul Molu, Sevda Korkmaz

**Affiliations:** 1 Department of Cardiology, School of Medicine, Firat University, 23119 Elazig, Turkey; hkorkmaz@firat.edu.tr; 2 Clinic of Cardiology, Gaziantep City Hospital, 27470 Gaziantep, Turkey; irfanyaman89@gmail.com; 3 Clinic of Cardiology, Battalgazi State Hospital, 44320 Malatya, Turkey; guneysarioglu@outlook.com; 4 Department of Psychiatry, School of Medicine, Firat University, 23119 Elazig, Turkey; esengulmez26@gmail.com (E.M.); skorkmaz@firat.edu.tr (S.K.)

**Keywords:** psychological hardiness, coronary artery ectasia, stressful life events

## Abstract

*Background and Objectives:* Coronary artery ectasia (CAE) is defined as a localized or diffuse dilatation of the coronary arteries and is often associated with atherosclerosis, congenital factors, or inflammatory conditions. Given emerging evidence that psychological factors may influence cardiovascular health, this study investigated whether psychological hardiness and past traumatic experiences are associated with CAE. *Materials and Methods:* This study employed a retrospective, cross-sectional, observational design involving 80 participants. All participants were administered a socio-demographic data form, the Beck Depression Inventory (BDI), the Beck Anxiety Inventory (BAI), the Personal Views Scale III-R (PVS III-R), and the Stressful Life Events Screening Form (SLESF). *Results:* The PVS III-R scale score for patients with CAE was significantly higher than that of the control group. The SLESF scores were markedly lower in the CAE group when compared to the control group, demonstrating that the control group experienced substantially more intense traumatic events when compared to CAE patients. Binary logistic regression was applied to determine variables associated with CAE, and the results were statistically significant. Notably, higher self-reported PVS III-R scores were independently associated with the presence of CAE. *Conclusions:* This study may provide a preliminary psychosomatic perspective on the pathophysiology of CAE, emphasizing the complexities of mind–body interactions. It suggests that high psychological hardiness may be associated with unexpected biological outcomes and warrants further investigation.

## 1. Introduction

Coronary artery ectasia (CAE), characterized by diffuse or regional dilatation of the coronary arteries, was defined as a 1.5- to 2-fold enlargement of at least one coronary artery compared with the adjacent normal coronary artery [1]. Post-coronary angiography CAE was reported to occur in 1.4% of cases by Hartnell et al. [2]. CAE is a common condition with various causes, and its management depends on symptom severity and anatomical structure [3]. The most common cause of CAE is atherosclerosis [4]. The etiology of CAE includes connective tissue diseases, infectious and inflammatory diseases, genetic causes, traumatic and toxic factors [5]. Destruction of muscle and elastic fibers in the vessel’s middle layer and disruption of collagen and elastin fibers are the main mechanisms of CAE [6].

However, it is known that certain personality traits and psychological factors could also significantly affect the development of heart diseases [7].

Psychological resilience is the ability to maintain mental health and adapt positively in the face of adversity. Resilience is not only an individual characteristic; it is a dynamic process that emerges with the interaction of biological, psychological, social, and environmental factors [8].

Our clinical observations had revealed that the psychological hardiness and response to stressful life events were different in patients with CAE when compared to patients with typical coronary angiography. Depression and anxiety, while well-known psychological factors in cardiovascular diseases, represent a multi-systemic structure akin to a personality trait that can capture different aspects of “resilience,” a dynamic adaptation process that can capture different aspects of psychosomatic vulnerability and adaptation, is a multi-systemic structure similar to a personality trait, and may have unique physiological correlations, such as autonomic regulation related to the non-occlusive nature of CAE.

Thus, the present study aimed to determine whether past traumatic events in life and psychological hardiness correlated with CAE. We also aimed to compare the levels of psychological hardiness as measured by the Personal Views Scale III-R (PVS III-R) in patients with CAE and those with normal coronary angiography findings after experiencing stress.

## 2. Materials and Methods

Participants were individuals who underwent coronary angiography after being assessed as high risk by the European Society of Cardiology Guidelines for the management of chronic coronary syndromes based on the results of a symptom-limited treadmill Exercise Stress Test performed according to the standard Bruce protocol at our hospital’s cardiology service over the past year. Coronary angiography was performed using 6-French right- and left-heart catheters and a Philips Medical Systems Allura Clarity angiography system with the Judkins technique. The first 40 patients with CAE who met the study criteria and agreed to participate were included in the patient group. The patient group included patients with only isolated CAE and no atherosclerotic plaques that led to more than 20% obstruction in the coronary arteries. The control group was composed of individuals with normal coronary arteries. The images of all patients with CAE were examined by 2 specialists. Coronary artery diameter was measured quantitatively based on the largest segment diameter with the diameter measurement feature of the angiography device. CAE was described as a 1.5–2-fold enlargement when compared to the diameter of the adjacent normal coronary artery. Also, the first 40 individuals who underwent coronary angiography in the cardiology clinic, met the study criteria, exhibited normal coronary vessels, and volunteered to participate in the study were included in the control group. In both the patient and the control groups, inclusion criteria also required the absence of any known psychiatric disease. After the participants completed the socio-demographic data form, the Beck Depression Inventory (BDI), the Beck Anxiety Inventory (BAI), the Stressful Life Events Screening Form (SLESF), and the Psychological Views Scale were applied to collect the study data. The research was carried out in accordance with the Declaration of Helsinki and received approval from the local ethics committee. Written and signed consent was obtained from each participant.

### 2.1. Data Collection Instruments

BDI: The inventory was developed by Beck in 1961 to measure the risk of depression, the variation in severity, and the level of depressive symptoms in adults [9]. It was determined that the scale cut-off point was 17. The 21-item Likert-type self-assessment scale has been frequently used in studies on depression. Each item measures a behavioral trait associated with depression. Items are scored on a scale of 0 to 3 based on the severity of depression. The total score ranges from 0 to 63. A score between 0 and 9 reflects no depressive symptoms, 10–16 indicates mild, 17–24 moderate, and 25 and above severe depressive symptoms.

BAI: BAI is a self-assessment scale developed by Beck et al. that measures the frequency of anxiety symptoms [10]. It is a Likert-type scale with 21 items, scored on a 0–3 scale. A high total score indicates a high anxiety level.

PVS III-R: The Personal Views Scale–III R was developed by Maddi and Khoshaba and comprises 18 items that reflect individual beliefs about oneself and life, organized into three factors: attachment, control, and challenge [11]. The scale is a 4-point Likert-type scale scored between 0 and 3 (0: not at all true, 1: somewhat true, 2: mostly true, and 3: quite true). The hardiness scale includes direct and inverse statements. Items 3, 4, 6, 8, 10, and 11 are reverse-scored on the scale. The three sub-dimensions are scored separately to obtain the total psychological hardiness score. Higher scores indicate greater psychological hardiness. In theoretical models, hardiness is defined as a personality trait/protective factor that fosters psychological resilience [12].

SLESF: The Stressful Life Events Screening Form, developed by Stamm and Rudolph, is a self-assessment scale that measures the severity of significant life events [13]. It is a 20-item 10-point Likert-type scale (“I never experienced” and “it is my experience”). Our scoring methodology is based on the study by Kessler et al. [14]. In this study, each item can be assigned a score of 0 (indicating that it does not describe the experience) or 1 (indicating that it accurately describes the experience). A general cut-off point of 3 aligns with the incidence figures reported in Kessler’s National Comorbidity Study. Consequently, scores of 3 or below are categorized as 0, while scores of 4 and above are categorized as 1.

For the SLESF, no formally validated Turkish adaptation was available; therefore, we used a translated version based on the original English items and the scoring approach described by Kessler et al. [14]. The BDI, BAI, and PVS III-R were used in their validated Turkish versions, which have demonstrated acceptable psychometric properties in Turkish populations [15,16,17].

### 2.2. Human Ethics and Consent to Participate Declarations

The local ethics committee of the Firat University School of Medicine examined and approved the detailed protocol for the current study (Approval Date/No.: 01.03.2018/05-01). Volunteering and signing the written consent form were required for participation in the study, both in the healthy control group and in patients with adjustment disorders. Particular attention was paid to confidentiality throughout the study procedures for both the patient and healthy control groups, and this was emphasized to the study subjects. Ethical principles were meticulously followed throughout the study, and all procedures were conducted in accordance with the 1983 version of the Declaration of Helsinki.

### 2.3. Statistical Analyses

The data were analyzed using SPSS software (IBM SPSS Statistics for Windows, Version 22.0, Armonk, NY, USA, IBM Corp.). By examining the data in their original form, we avoided any possible inaccuracies that could arise from imputing missing values. Descriptive analysis was performed to characterize the study population. The normality of the variables was assessed using the Kolmogorov–Smirnov test. For pairwise comparisons, the Independent-Samples *t*-test was used for normally distributed data, and the Mann–Whitney U test was used for non-normally distributed data. We set the threshold for statistical significance at *p* < 0.05 in this study. Additionally, a 95% confidence interval that did not include zero was considered statistically significant. Categorical variables were analyzed with the appropriate chi-square test and reported as percentages (%) and absolute counts. Means and standard deviations were utilized to present continuous variables for normally distributed data, whereas medians (interquartile range) were used for non-normally distributed data. The relationship between continuous variables was assessed using Pearson correlation for normally distributed variables and Spearman correlation for non-normally distributed variables. Furthermore, Binary logistic regression analysis was performed to identify variables associated with CAE. The effect size of the study was evaluated using Cohen’s d.

## 3. Results

Sociodemographic data for the patient and control groups are presented in Table 1. Independent-samples *t*-tests indicated significant differences between the two groups on both the SLESF and the PVS III-R. The group with CAE demonstrated significantly lower SLESF scores. Higher scores on the PVS III-R than in the other group were revealed by an independent-samples *t*-test. No notable differences were observed in BDI or BAI scores or in age between the two groups (Table 2). There was no significant difference in intergroup trauma counts (*p* = 0.834). The three most frequently described traumatic events included natural disasters, the death of a friend, or the diagnosis of a relative or friend with a life-threatening disease in both groups (Table 3). Group comparisons indicated that participants in the control group reported significantly greater exposure to certain types of traumatic events. (Table 4). Effect size analyses indicated that the differences in total SLESF scores between groups were of medium magnitude (Cohen’s d = 0.455, 95% CI (0.010, 0.898)). Conversely, PVS III-R scores exhibited a medium-sized effect in the opposite direction (Cohen’s d = −0.455, 95% CI (−0.898, −0.010)). Pearson correlation analyses revealed a strong positive correlation between depression and anxiety levels (r = 0.633, *p* < 0.001). In addition, depressive symptoms were weakly but significantly associated with stressful life events (r = 0.271, *p* = 0.015).

**Figure 1:**
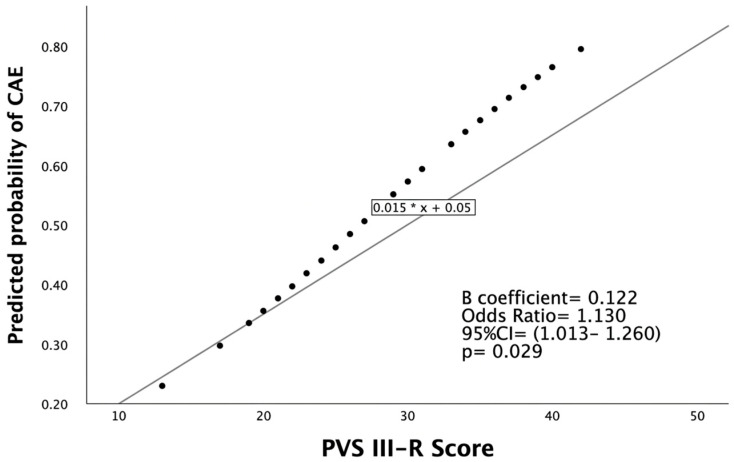
The logistic regression model estimates the log-odds of CAE and does not imply linear changes in probability across the PVS III-R score range. PVS = Personal Views Scale, CI = Confidence Interval, CAE = coronary artery ectasia.

A Binary logistic regression analysis was performed to identify variables associated with CAE. The model was statistically significant (Omnibus Test of Model Coefficients: χ^2^ (7) = 15.793, *p* = 0.027), indicating that the set of variables reliably distinguished between the presence and absence of CAE (Table 5). The overall model fit was acceptable, with Nagelkerke R^2^ = 0.242 and Cox & Snell R^2^ = 0.181, indicating that approximately 24% of the variance in the presence of CAE was explained by the model. The Hosmer-Lemeshow goodness-of-fit test was not significant (*p* = 0.058), indicating a good fit between the observed and expected values. Among variables, the PVS III-R score was statistically significant (B = 0.122, OR = 1.130, 95% CI (1.013–1.260), *p* = 0.029). This indicates that higher hardiness scores were associated with increased odds of CAE (Figure 1).

The calculated statistical power was about 0.71. This level suggests a moderate ability to identify the observed association. While it is enough to find medium effect sizes, the study may not have enough power to reliably detect small effects. The moderate power points to the need for larger prospective studies to confirm these results.

## 4. Discussion

To our knowledge, our study is the first to investigate the relationship among psychological hardiness, stressful life events, and CAE. In the study, although the number of traumatic events did not differ between groups, the perceived severity (SLESF score) was lower in the CAE. That is, individuals with CAE reported experiencing these stressors with less severity compared to their counterparts. Furthermore, their psychological hardiness was greater than that of the control group. The analysis comparing patients with CAE to the control group indicated that there were no significant differences in their depression and anxiety scores.

In recent years, several studies have investigated the relationship between CAE and psychological states and diseases. Gürbüz AS et al. found that depression scores in patients with CAE were significantly higher than those in individuals with normal coronary arteries [18]. Ozturk et al. found that patients with CAE demonstrate significantly lower scores for anxiety and depression when compared to patients with coronary artery disease alone [19].

The PVS III-R scale is among the most frequently used and appropriate measures of psychological hardiness in the general population [20]. Personality hardiness, defined by commitment, control, and challenge, enables individuals to manage stressors and turn them into opportunities for growth, thereby improving their resilience across different stressful circumstances [21]. In light of this information, we evaluated psychological hardiness indirectly using the PVS III-R. This scale measures hardiness, a construct closely associated with adaptive functioning under stress, and is recognized as a reliable predictor of resilience.

Traumatic events lead to negative experiences. However, sometimes, it is possible for individuals who were exposed to traumatic events to prevent the negative consequences of the trauma and come out of it stronger. These positive effects are described as “perceived benefits”, “stress-induced growth”, or “post-traumatic growth” [22,23]. For post-traumatic growth, the event should be shocking, and the individual should fight against this trauma [24]. However, post-traumatic growth and strength are not observed in every individual and at the same rate. Because certain environmental factors, such as the level of stress induced by the trauma, social support, sociocultural effects, and individual traits, such as personality, stress management, coping mechanisms, and emotional self-disclosure, could affect post-traumatic growth [25,26].

There is also a close relationship between post-traumatic strength and psychological resilience. Because the psychological resilience induced by a traumatic experience contributes to an individual’s ability to cope with subsequent negative experiences. Individuals who are able to recover from difficulties and traumatic experiences at different stages in life, cope with these challenges, and maintain their vital functions through adaptation are described as psychologically resilient [27,28]. It has been assumed that those with high resilience are curious, active, and have a sense of control over their lives [20]. It is also known that individuals with high psychological resilience can cope with stressful events and easily adapt to their environment during challenges [29]. Past traumatic experiences could lead to certain physical and psychological disorders [30]. It is also known that the adverse effects of traumatic experiences in childhood are not limited to childhood but also adulthood [28]. Traces of the traumatic experiences could also be activated by a new traumatic experience, triggering a novel psychiatric or psychosomatic presentation [31,32,33].

Residual confounding by unmeasured or insufficiently modeled cardiovascular, inflammatory, socioeconomic, or behavioral variables cannot be excluded. Although classical cardiovascular risk factors were similar between groups, the limited number of events per variable precluded inclusion of all potential covariates in the logistic regression model. Therefore, psychological hardiness may act as a surrogate marker of broader biopsychosocial profiles rather than as an independent etiological determinant.

In interpreting our findings, it is important to note that higher PVS III-R scores may not reflect solely genuine adaptive resilience. Alternative explanations include repressive coping, broader patterns of emotional suppression, or culturally shaped reporting norms that discourage the open expression of distress. Self-report indices of resilience and emotion regulation can conflate flexible, adaptive coping with defensive inhibition of negative affect, and the psychological meaning of strategies such as expressive suppression varies across cultural contexts. Thus, elevated PVS III-R scores in our sample should be interpreted cautiously, as they may partly capture context-specific norms of ‘staying strong’ or socially desirable responding, rather than uniformly indicating positive adaptation [34,35].

Our results can be interpreted in the context of more general psychosocial and psychophysiological models that propose the coexistence of what appears to be high resilience with high levels of physiological stress burden in some individuals, rather than resilience being a marker of adaptive functioning per se. High effort coping styles such as John Henryism have been associated with high allostatic load despite low levels of depressive symptoms, reflecting a trade-off between psychological adaptation and multisystem biological “wear and tear”. Allostatic load refers to the theoretical cumulative effects of chronic stress on the cardiovascular, metabolic, immune, and neuroendocrine systems and has been consistently shown to be associated with adverse physical and mental health outcomes, including mood disorders and mortality. In this context, autonomic dysregulation and psychosomatic symptoms may reflect the biological “costs” of effortful coping and stress exposure, even when self-reported resilience or functioning is intact. In keeping with these findings, our data are consistent with the hypothesis that higher levels of self-reported resilience in some individuals may be a marker of underlying biological strain, although they do not provide support for causal inferences and should be considered a tentative hypothesis requiring longitudinal validation [36,37,38].

Biological, genetic, and lifestyle determinants are likely to account for a substantially larger share of variance in vulnerability and course than psychosocial factors alone, as suggested by evidence on age-related neurobiological changes, cardiometabolic and inflammatory pathways, and non-genetic risk factors across mental disorders. In this context, our results are best understood as adding a psychosomatic layer—emphasizing stress-related autonomic, neuroendocrine, and lifestyle-linked processes—within a broader biopsychosocial framework, rather than providing a comprehensive explanatory model of these conditions [39].

Low psychological resilience and coping skills in traumatic experiences affect physical processes and could become a chronic stress response. Cognitive-emotional-physical structures are fully activated by an event that an individual perceives as a threat [40,41]. This could lead to certain negative personal consequences, such as high health-compromising behaviors and adaptation problems, as well as illness and disability, even premature death [42].

Possible hypothetical mechanisms that could explain the results: (The following mechanisms are purely hypothetical and are presented as conceptual models to guide future research; they are not directly supported by biological measurements in the present study.)

(1) Chronic low-level inflammation and vascular remodeling: Resilience protects against emotional distress after stressful events and failure. [43]. However, this does not mean stress’s physiological effects are gone. Someone might believe they are managing stress, but their body could stay in a low-level “fight or flight” mode. Chronic stress can induce low-grade inflammation by altering tissue sensitivity to anti-inflammatory signals from the hypothalamus–pituitary–adrenal axis and the autonomic nervous system. [44]. This inflammatory process releases enzymes, especially matrix metalloproteinases, which degrade elastin and collagen in the vessel’s tunica media. This destruction causes vessel dilation and ectasia. [45].

(2) Autonomic Nervous System Dysfunction and Nitric Oxide (NO) Pathway: Combined psychological and biophysiological features, such as high vagal activity during stress and the restoration of cortisol/DHEA balance after stress, predict psychological resilience [46]. These individuals may cope with stress through sustained parasympathetic activation or a unique balance of sympathetic and parasympathetic systems. Acetylcholine, a key parasympathetic neurotransmitter, stimulates Nitric Oxide release from endothelial cells, which acts as a potent vasodilator [47]. This, NO–cGMP signaling is increasingly recognized as a central modulator of structural remodeling in pulmonary and systemic vascular beds, linking chronic alterations in autonomic balance to changes in vascular wall architecture [48]. These putative mechanisms should be regarded as hypothesis-generating and require dedicated, longitudinal, and biomarker-informed studies.

The primary limitation is the study’s retrospective, cross-sectional design, which, while effective for demonstrating an association, precludes any inference of causality. There is potential selection bias arising from the inclusion of patients who underwent coronary angiography. It is unclear whether high hardiness is a pre-existing trait that contributes to the pathophysiology of CAE or a psychological adaptation that develops in response to the condition. Lower perceived severity of events could be due to under-reporting or to differences in appraisal. Prospective longitudinal studies are essential to disentangle this complex relationship. Second, the study was conducted at a single center with a modest sample size (N = 80), which may restrict the generalizability of our findings. In logistic regression, a Nagelkerke R^2^ of 0.242 indicates modest explanatory power. However, psychological factors may be only one component of the multifactorial pathophysiology of CAE. The use of convenience sampling could also introduce unforeseen selection biases. Residual confounding by unmeasured or insufficiently modeled cardiovascular variables (e.g., inflammatory markers, BMI, coronary dominance) cannot be excluded. Cardiovascular risk factors are important confounding factors in CAE. In our sample, hypertension, diabetes, hyperlipidemia, and smoking were similar across groups, but due to the limited number of events per variable, we did not include all of them in the logistic model to prevent overfitting. Any potential link between hardiness-related traits and CAE is likely embedded within a broader network of biological, behavioral, and socioeconomic risk factors, which we were not able to fully control for in this study.

Future large-scale, multicenter, and longitudinal studies are needed to determine the direction of association between CAE and psychological hardiness and to validate our results across more diverse populations. An additional limitation concerns temporal ambiguity. Because of the cross-sectional design, it is unclear whether higher PVS III-R scores preceded the development of CAE or emerged as a psychological adaptation following diagnosis. It is possible that individuals with CAE engage in cognitive reframing (‘staying strong’) that inflates self-reported hardiness, thereby implying reverse causation. Longitudinal studies are needed to clarify the temporal direction of the association.

Third, our assessment of psychological constructs relied exclusively on self-report questionnaires. SLESF binary coding may lead to a potential loss of sensitivity; however, Cronbach’s alpha, a reliability coefficient, is not available for this sample. These instruments are susceptible to recall bias, particularly for retrospective accounts of stressful life events, and to social desirability bias. Furthermore, although our findings point towards a mechanism related to post-traumatic growth, we did not employ a specific instrument to measure this phenomenon, representing a missed opportunity for a more nuanced psychological characterization. Fourthly, while we utilized validated versions of the scales, we did not conduct a reliability analysis on the study sample. Consequently, the psychometric performance of these instruments in our specific population has not yet been established. Finally, and perhaps most importantly, while our discussion proposes potential pathophysiological mechanisms involving chronic inflammation and autonomic nervous system dysfunction, the study lacked direct measurement of biological correlates.

Another important limitation concerns the assessment of stressful life events. Although we followed the original structure and scoring rules of the SLESF, a fully validated Turkish version of this instrument was not available. Consequently, potential issues related to translation, cultural equivalence, and measurement error may have affected the accuracy and comparability of SLESF scores in our sample, and the observed associations involving stressful life events should be interpreted with particular caution.

Moreover, we conducted multiple comparisons across SLESF subdomains and other psychological variables without formal correction for multiple testing. In this context, the borderline *p*-values (e.g., *p* ≈ 0.04–0.05) and medium effect sizes with wide confidence intervals increase the risk of Type I error. Therefore, the present findings should be interpreted as exploratory and hypothesis-generating rather than definitive.

It is important to emphasize that the PVS III-R measures self-reported psychological hardiness rather than objectively validated adaptive resilience. Elevated scores may reflect multiple psychological processes, including adaptive coping, defensive emotional suppression, or culturally shaped norms regarding the expression of distress. Therefore, the construct captured in this study should be interpreted as a proxy psychological variable rather than a direct measure of functional or biological resilience.

## 5. Conclusions

This study is the first to suggest an exploratory association between CAE and higher levels of self-reported psychological hardiness. Our findings indicated that individuals with CAE, despite encountering a similar number of traumatic events, perceived these experiences as less severe and exhibited statistically significantly higher levels of psychological hardiness compared to the control group.

These findings might challenge the prevailing belief that psychological resilience is universally protective. We propose the hypothesis that, while resilience may mitigate the acute emotional impacts of stress, it could also obscure or predispose individuals to maladaptive physiological responses, such as chronic low-grade inflammation or dysfunction of the autonomic nervous system. In the long run, these responses could be involved in vascular remodeling processes potentially related to CAE, although this remains speculative. The absence of significant differences in depression and anxiety scores between the groups is consistent with, but does not prove, our hypothesis that the observed association may not be fully explained by current mood states, but could instead reflect a more stable, trait-like construct of resilience.

The cross-sectional design of our study limits our ability to establish causality. To validate these findings and elucidate the underlying psycho-biological mechanisms, future research should utilize large, multicenter, prospective designs that assess inflammatory markers, such as Matrix Metalloproteinases, along with indicators of autonomic function. Ultimately, our study provides a novel psychosomatic perspective on the pathophysiology of CAE and highlights the intricacies of the mind–body interaction, suggesting that high psychological hardiness may be associated with unexpected biological outcomes.

Given the limited sample size and multiple testing without adjustment, our results should be interpreted with caution as preliminary, hypothesis-generating evidence. Future longitudinal research is required to determine whether hardiness-related traits represent a risk factor for, correlate of, or consequence of CAE.

## Figures and Tables

**Table 1:** Patient and control group demographics.

	Ectasia Group (n = 40)		Control Group (n = 40)	
	n	%	n	%
Sex (female)	17	42.5	18	45
Smokers	14	35	10	25
Diabetes mellitus	7	17.5	8	20
Hypertension	15	37.5	13	32.5
Hyperlipidemia	14	35	12	30
Liquor use	1	2.5	5	12.5
Concomitant disease	22	55	16	40
Presence of heart disease in the family	19	47.5	19	47.5
Death in the family due to a heart disease	14	35	13	32.5
Marital status (married)	37	92.5	38	95
Primary school graduate	28	70	32	80
Secondary school graduate	7	17.5	4	10
College graduate	5	12.5	4	10
Residence (urban)	34	85	26	65
Income level (low)	7	17.5	8	20
Prior psychiatric treatment	6	15	8	20

Categorical variables are demonstrated as numbers (n) and percentages (%).

**Table 2:** The comparison of ectasia and control group scale scores.

	Ectasia (n = 40)	Control (n = 40)	*p*	t
Age (years) (SD)	60.95 (10.8)	58.5 (12.4)	0.353	0.936
SLESF score (SD)	17.8 (19.2)	28.0 (25)	0.045 *	−2.036
PVS III-R score (SD)	28.0 (5.6)	25.6 (4.97)	0.045 *	2.035
Trauma count (SD)	3.88 (5.2)	4.1 (4.3)	0.834	−0.211
BDI score (SD)	11.68 (8.5)	12.08 (9.04)	0.839	−0.204
BAI score (SD)	14.3 (9.92)	14.1 (12.3)	0.920	0.100

Normally distributed continuous variables are expressed as mean ± standard deviation (SD). Differences between groups were analyzed using an independent-samples *t*-test. Abbreviations: SLESF: Stressful Life Events Screening Form, PVS: Personal Views Scale, BDI: Beck Depression Inventory, BAI: Beck Anxiety Inventory. * *p* < 0.05.

**Table 3:** Comparison of stressful life events experienced between groups.

	Ectasia (n = 40)	Control (n = 40)	*p*
Natural disaster n (%)	20 (50)	31 (77.5)	0.019 *
Plane crash/industrial disaster n (%)	5 (12.5)	6 (15)	1.000
Serious accident n (%)	12 (30)	13 (32.5)	1.000
Radiation n (%)	4 (10)	2 (5)	0.675
Illness (relative/close friend) n (%)	14 (35)	16 (40)	0.818
Partner-child death n (%)	8 (20)	6 (15)	0.770
Death of a friend n (%)	17 (42.5)	25 (62.5)	0.117
Kidnapping/hostage n (%)	2 (5)	4 (10)	0.675
Terror/torture n (%)	5 (12.5)	5 (12.5)	1.000
War/conflict n (%)	5 (12.5)	4 (10)	1.000
Seeing a corpse n (%)	7 (17.5)	8 (20)	1.000
Severe injury n (%)	3 (7.5)	3 (7.5)	1.000
Armed assault n (%)	5 (12.5)	4 (10)	1.000
Beating in childhood n (%)	8 (20)	7 (17.5)	1.000
Beating in adulthood n (%)	8 (20)	5 (12.5)	0.546
Witnessing beating n (%)	10 (25)	10 (25)	1.000
Sexual abuse in childhood n (%)	5 (12.5)	3 (7.5)	0.712
Sexual abuse in adulthood n (%)	4 (10)	2 (5)	0.675
Witnessing sexual abuse n (%)	4 (10)	2 (5)	0.675
Stressful event n (%)	10 (25)	8 (20)	0.790

Categorical variables are demonstrated as numbers (n) and percentages (%). * *p* < 0.05.

**Table 4:** Intergroup comparison of stressful life event scores.

	Ectasia (n = 40)	Control (n = 40)	*p*	t
Natural disaster n (SD)	3.13 (3.93)	6.53 (4.09)	0.001 *	−3.792
Plane crash/industrial disaster n (SD)	0.33 (1.18)	0.98 (2.61)	0.155	−1.436
Serious accident n (SD)	1.55 (2.99)	1.45 (2.82)	0.878	0.154
Radiation n (SD)	0.23 (0.8)	0.13 (0.65)	0.541	0.614
Illness (relative/close friend) n (SD)	2.35 (3.66)	2.7 (3.7)	0.672	−0.426
Partner-child death n (SD)	1.48 (3.45)	1.18 (2.99)	0.679	0.415
Death of a friend n (SD)	2.53 (3.89)	4.9 (4.35)	0.013 *	−2.547
Kidnapping/hostage n (SD)	0.05 (0.22)	0.95 (2.63)	0.034 *	−2.156
Terror/torture n (SD)	0.55 (1.87)	0.73 (2.15)	0.698	−0.389
War/conflict n (SD)	0.53 (1.75)	0.68 (2.26)	0.741	−0.332
Seeing a corpse n (SD)	0.83 (2.48)	1.43 (3.2)	0.349	−0.943
Severe injury n (SD)	0.05 (0.22)	0.2 (0.85)	0.285	−1.076
Armed assault n (SD)	0.15 (0.66)	0.68 (2.33)	0.173	−1.374
Beating in childhood n (SD)	0.86 (2.13)	1.13 (2.62)	0.402	−0.842
Beating in adulthood n (SD)	1.08 (2.85)	0.68 (2.01)	0.470	0.726
Witnessing beating n (SD)	1.28 (2.76)	1.58 (3.17)	0.653	−0.451
Sexual abuse in childhood n (SD)	0.05 (0.22)	0.58 (1.93)	0.092	−1.706
Sexual abuse in adulthood n (SD)	0.05 (0.22)	0.35 (1.61)	0.247	−1.168
Witnessing sexual abuse n (SD)	0.05 (0.22)	0.25 (1.13)	0.274	−1.102
Stressful event n (SD)	0.9 (2.44)	0.73 (1.68)	0.709	0.374
Total score n (SD)	17.8 (19.2)	28.0 (25.2)	0.045 *	−2.036

Normally distributed continuous variables are expressed as mean ± standard deviation (SD). Differences between groups were analyzed using an independent-samples *t*-test. * *p* < 0.05.

**Table 5:** Logistic regression analysis to determine the variables associated with coronary ectasia.

Variables	B	SE	Wald χ^2^	df	Exp(B)	95% CI for Exp(B)	*p*
Age	0.040	0.024	2.638	1	1.040	0.992–1.091	0.104
Smoking	0.774	0.633	1.494	1	2.168	0.627–7.498	0.222
Alcohol consumption	−2.626	1.373	3.657	1	0.072	0.005–1.068	0.056
BDI score	0.001	0.038	0.000	1	1.001	0.929–1.079	0.983
BAI score	−0.007	0.030	0.054	1	0.993	0.937–1.053	0.816
PVS III-R score	0.122	0.056	4.786	1	1.130	1.013–1.260	0.029 *
SLESF score	−0.023	0.013	3.408	1	0.977	0.953–1.001	0.065

Alcohol consumption, gender and smoking are presented as dummy variables. 95% CI for Exp(B) presented as lower and upper limits. Nagelkerke R Square: 0.242. Abbreviations: PVS: Personal Views Scale, BDI: Beck Depression Inventory, BAI: Beck Anxiety Inventory * *p* < 0.05.

## Data Availability

The data presented in this study are available on request from the corresponding author.

## Abbreviations

The following abbreviations are used in this manuscript:

CAE	Coronary artery ectasia
BDI	Beck depression inventory
BAI	Beck anxiety inventory
PVS III-R	Personal views scale III-R
SLESF	Stressful life events screening form
NO	Nitric oxide
DHEA	Dehydroepiandrosterone

## References

[1] Swaye P.S., Fisher L.D., Litwin P., Vignola P.A., Judkins M.P., Kemp H.G., Mudd J.G., Gosselin A.J. (1983). Aneurysmal coronary artery disease. Circulation.

[2] Hartnell G.G., Parnell B.M., Pridie R.B. (1985). Coronary artery ectasia: Its prevalence and clinical significance in 4993 patients. Br. Heart J..

[3] Woźniak P., Iwańczyk S., Błaszyk M., Stępień K., Lesiak M., Mularek-Kubzdela T., Araszkiewicz A. (2024). Coronary Artery Aneurysm or Ectasia as a Form of Coronary Artery Remodeling: Etiology, Pathogenesis, Diagnostics, Complications, and Treatment. Biomedicines.

[4] Roberts W.C. (2011). Natural history, clinical consequences, and morphologic features of coronary arterial aneurysms in adults. Am. J. Cardiol..

[5] Díaz-Zamudio M., Bacilio-Pérez U., Herrera-Zarza M.C., Meave-González A., Alexanderson-Rosas E., Zambrana-Balta G.F., Kimura-Hayama E.T. (2009). Coronary artery aneurysms and ectasia: Role of coronary CT angiography. Radiographics.

[6] Antoniadis A.P., Chatzizisis Y.S., Giannoglou G.D. (2008). Pathogenetic mechanisms of coronary ectasia. Int. J. Cardiol..

[7] Sirri L., Fava G.A., Guidi J., Porcelli P., Rafanelli C., Bellomo A., Grandi S., Grassi L., Pasquini P., Picardi A. (2012). Type A behaviour: A reappraisal of its characteristics in cardiovascular disease. Int. J. Clin. Pract..

[8] Masten A.S., Lucke C.M., Nelson K.M., Stallworthy I.C. (2021). Resilience in Development and Psychopathology: Multisystem Perspectives. Annu. Rev. Clin. Psychol..

[9] Beck A.T., Ward C.H., Mendelson M., Mock J., Erbaugh J. (1961). An Inventory for Measuring Depression. Arch. Gen. Psychiatry.

[10] Beck A.T., Epstein N., Brown G., Steer R.A. (1988). An Inventory for Measuring Clinical Anxiety: Psychometric Properties. J. Consult. Clin. Psychol..

[11] Maddi S.R., Khoshaba D.M., Persico M., Lu J., Harvey R., Bleecker F. (2002). The personality construct of hardiness: II. Relationships with comprehensive tests of personality and psychopathology. J. Res. Personal..

[12] Chmitorz A., Kunzler A., Helmreich I., Tüscher O., Kalisch R., Kubiak T., Wessa M., Lieb K. (2018). Intervention studies to foster resilience—A systematic review and proposal for a resilience framework in future intervention studies. Clin. Psychol. Rev..

[13] Stamm B., Rudolph J., Stamm B. (1996). Psychometric review of Stressful Life Experiences Screening. Measurement of Stress, Trauma and Adaptation.

[14] Kessler R.C., Sonnega A., Bromet E., Hughes M., Nelson C.B. (1995). Posttraumatic Stress Disorder in the National Comorbidity Survey. Arch. Gen. Psychiatry.

[15] Ozdemir H.D., Dagdeviren H.N. (2024). Construct validity study of the Turkish form of the Short Beck Depression Inventory. Eurasian J. Fam. Med..

[16] Çınaroğlu M., Yılmazer E., Sayar G.H. (2025). Psychological impact of the 2023 Kahramanmaraş earthquakes on non-victims: A cross-sectional study. BMC Public Health.

[17] Durak M. (2002). Deprem Yaşamış Üniversite Öğrencilerinin Psikolojik Belirtilerini Yordamada Psikolojik Dayanıklılığın Rolü. Yayımlanmamış Yüksek Lisans Tezi.

[18] Gürbüz A.S., Alsancak Y., Saklı B., Düzenli M.A. (2019). Association between depression and anxiety scores and inflammation in patients with isolated coronary artery ectasia. Turk Kardiyol. Dern. Ars..

[19] Ozturk S., Yalvac H., Sivri N., Ozturk H., Kılıc Y., Bulut E., Celik A., Barlas Y., Tengiz I., Yetkin E. (2015). Anxiety and depression scores in patients with coronary artery disease and coronary artery ectasia. Int. J. Cardiol..

[20] Sharif Nia H., Froelicher E.S., Hosseini L., Ashghali Farahani M. (2022). Evaluation of Psychometric Properties of Hardiness Scales: A Systematic Review. Front. Psychol..

[21] Maddi S.R. (2005). On hardiness and other pathways to resilience. Am. Psychol..

[22] Dürü Ç. (2014). Travma sonrası büyüme. Ufuk Üniversitesi Sos. Bilim. Enstitüsü Derg..

[23] Inci F., Boztepe H. (2013). Post traumatic growth: If something not killing could be strengthned?. J. Psychiatr. Nurs..

[24] Tedeschi R.G., Park C.L., Calhoun L.G. (1998). Posttraumatic Growth: Positive Changes in the Aftermath of Crisis.

[25] Ramos C., Leal I. (2013). Posttraumatic Growth in the Aftermath of Trauma: A Literature Review About Related Factors and Application Contexts. Psychol. Community Health.

[26] Calhoun L.G., Tedeschi R.G. (2014). The foundations of posttraumatic growth: An expanded framework. Handbook of Posttraumatic Growth.

[27] Masten A.S. (2014). Global Perspectives on Resilience in Children and Youth. Child Dev..

[28] Luthar S.S., Cicchetti D., Becker B. (2000). The construct of resilience: A critical evaluation and guidelines for future work. Child Dev..

[29] Judkins S., Rind R. (2005). Hardiness, job satisfaction, and stress among home health nurses. Home Health Care Manag. Pract..

[30] Rutter M. (1987). Psychosocial Resilience and Protective Mechanisms. Am. J. Orthopsychiatry.

[31] Sar V., Ozturk E. (2006). What is trauma and dissociation?. J. Trauma Pract..

[32] Dube S.R., Fairweather D., Pearson W.S., Felitti V.J., Anda R.F., Croft J.B. (2009). Cumulative childhood stress and autoimmune diseases in adults. Psychosom. Med..

[33] Häuser W., Kosseva M., Üceyler N., Klose P., Sommer C. (2011). Emotional, physical, and sexual abuse in fibromyalgia syndrome: A systematic review with Meta-Analysis. Arthritis Care Res..

[34] Takeuchi M., Matsunaga M., Miyake A., Egashira R., Hotta S., Nakano M., Moriguchi M., Yasuno F., Myowa M., Hagihara K. (2025). The validity of a new resilience scale: The Japan Resilience Scale (J-RS) for mothers with a focus on cultural aspects. BMC Public Health.

[35] Robinson G., Lee E., Leckning B., Silburn S., Nagel T., Midford R. (2022). Validity and reliability of resiliency measures trialled for the evaluation of a preventative Resilience-promoting social-emotional curriculum for remote Aboriginal school students. PLoS ONE.

[36] Robinson M.N., Thomas Tobin C.S. (2021). Is John Henryism a Health Risk or Resource?: Exploring the Role of Culturally Relevant Coping for Physical and Mental Health among Black Americans. J. Health Soc. Behav..

[37] Guidi J., Lucente M., Sonino N., Fava G.A. (2021). Allostatic Load and Its Impact on Health: A Systematic Review. Psychother. Psychosom..

[38] Bozzatello P., Marin G., Gabriele G., Brasso C., Rocca P., Bellino S. (2024). Metabolic Dysfunctions, Dysregulation of the Autonomic Nervous System, and Echocardiographic Parameters in Borderline Personality Disorder: A Narrative Review. Int. J. Mol. Sci..

[39] Szymkowicz S.M., Gerlach A.R., Homiack D., Taylor W.D. (2023). Biological factors influencing depression in later life: Role of aging processes and treatment implications. Transl. Psychiatry.

[40] Fergusson D.M., Horwood L.J., Luthar S.S. (2003). Resilience to Childhood Adversity: Results of a 21-Year Study. Resilience and Vulnerability: Adaptation in the Context of Childhood Adversities.

[41] Goldberger L., Breznitz S. (1993). Handbook of Stress: Theoretical and Clinical Aspects.

[42] Anda R.F., Dong M., Brown D.W., Felitti V.J., Giles W.H., Perry G.S., Valerie E.J., Dube S.R. (2009). The relationship of adverse childhood experiences to a history of premature death of family members. BMC Public Health.

[43] Johnson J., Panagioti M., Bass J., Ramsey L., Harrison R. (2017). Resilience to emotional distress in response to failure, error or mistakes: A systematic review. Clin. Psychol. Rev..

[44] Rohleder N. (2011). Variability in stress system regulatory control of inflammation: A critical factor mediating health effects of stress. Expert Rev. Endocrinol. Metab..

[45] Wang X., Khalil R.A. (2018). Matrix Metalloproteinases, Vascular Remodeling, and Vascular Disease. Adv. Pharmacol..

[46] Lau W.K.W., Tai A.P.L., Chan J.N.M., Lau B.W.M., Geng X. (2021). Integrative psycho-biophysiological markers in predicting psychological resilience. Psychoneuroendocrinology.

[47] Zuccolo E., Laforenza U., Negri S., Botta L., Berra-Romani R., Faris P., Scarpellino G., Forcaia G., Pellavio G., Sancini G. (2019). Muscarinic M5 receptors trigger acetylcholine-induced Ca^2+^ signals and nitric oxide release in human brain microvascular endothelial cells. J. Cell. Physiol..

[48] Carlström M., Weitzberg E., Lundberg J.O. (2024). Nitric Oxide Signaling and Regulation in the Cardiovascular System: Recent Advances. Pharmacol. Rev..

